# Air pollution and anemia as risk factors for pneumonia in ecuadorian children: a retrospective cohort analysis

**DOI:** 10.1186/1476-069X-10-93

**Published:** 2011-11-03

**Authors:** Aaron M Harris, Fernando Sempértegui, Bertha Estrella, Ximena Narváez, Juan Egas, Mark Woodin, John L Durant, Elena N Naumova, Jeffrey K Griffiths

**Affiliations:** 1 Tufts University School of Medicine, 136 Harrison Avenue, Boston, MA, USA; 2 Corporación Ecuatoriana de Biotecnología, Quito, Ecuador; 3 Medical School, Central University of Ecuador, Quito, Ecuador; 4 Tufts University School of Engineering, Medford, MA, USA; 5 Friedman School of Nutrition Science and Policy, Tufts University, Boston, MA, USA; 6 Cummings School of Veterinary Medicine, Grafton, MA, USA

## Abstract

**Background:**

Ambient air pollution and malnutrition, particularly anemia, are risk factors for pneumonia, a leading cause of death in children under five. We simultaneously assessed these risk factors in Quito, Ecuador.

**Methods:**

In 2005, we studied two socioeconomically similar neighborhoods in Quito: Lucha de los Pobres (LP) and Jaime Roldos (JR). LP had relatively high levels of air pollution (annual median PM_2.5 _= 20.4 μg/m^3^; NO_2 _= 29.5 μg/m^3^) compared to JR (annual median PM_2.5 _= 15.3 μg/m^3^; NO_2 _= 16.6 μg/m^3^). We enrolled 408 children from LP (more polluted) and 413 children from JR (less polluted). All subjects were aged 18-42 months. We obtained medical histories of prior physician visits and hospitalizations during the previous year, anthropometric nutrition data, hemoglobin levels, and hemoglobin oxygen saturation via oximetry.

**Results:**

In anemic children, higher pollution exposure was significantly associated with pneumonia hospitalization (OR = 6.82, 95%CI = 1.45-32.00; P = 0.015). In non-anemic children, no difference in hospitalizations by pollution exposure status was detected (OR = 1.04, NS). Children exposed to higher levels of air pollution had more pneumonia hospitalizations (OR = 3.68, 1.09-12.44; P = 0.036), total respiratory illness (OR = 2.93, 95% CI 1.92-4.47; P < 0.001), stunting (OR = 1.88, 1.36-2.60; P < 0.001) and anemia (OR = 1.45, 1.09-1.93; P = 0.013) compared to children exposed to lower levels of air pollution. Also, children exposed to higher levels of air pollution had significantly lower oxygen saturation (92.2% ± 2.6% vs. 95.8% ± 2.2%; P < 0.0001), consistent with air pollution related dyshemoglobinemia.

**Conclusions:**

Ambient air pollution is associated with rates of hospitalization for pneumonia and with physician's consultations for acute respiratory infections. Anemia may interact with air pollution to increase pneumonia hospitalizations. If confirmed in larger studies, improving nutrition-related anemia, as well as decreasing the levels of air pollution in Quito, may reduce pneumonia incidence.

## Background

Pneumonia is the leading cause of childhood death [1,2]. The pathogenesis of pneumonia includes environmental and host factors, such as air pollution and malnutrition [2]. Air pollutants are respiratory irritants and increase susceptibility to acute respiratory infections (ARI) [3,4]. Air pollution increases morbidity and mortality largely from respiratory diseases [5], and reducing ambient air pollution increases lifespan [6]. Particulate air pollution (PM), especially particles < 10 μm (PM_10_) and < 2.5 μm (PM_2.5_) in aerodynamic diameter, and nitrogen oxides (NO_x_) are specifically linked to childhood ARI [7-9]. Outdoor (ambient) air pollution, and indoor pollution from biofuel use, have been identified as major pneumonia risk factors [2,3].

Globally, many malnourished children are exposed to high levels of air pollution. Also, because malnourished children are known to be at increased risk for pneumonia [1,2,10,11], a synergistic relationship between ambient air pollution and malnutrition has been postulated [2]. Previous studies of pneumonia have examined either air pollution or malnutrition [e.g. [12-15]] but not both risk factors simultaneously. We explored the relationship between air pollution and malnutrition in Quito, Ecuador where both risk factors are prevalent. Our primary objective was to determine if interaction between malnutrition and air pollution was evident. To our knowledge this is the first study of possible interaction effects between ambient air pollution and malnutrition as risk factors for pneumonia in children.

## Methods

### Ethics Approval

This study was approved by the Tufts University Institutional Review Board and the Ethical Committee of the Corporación Ecuatoriana de Biotechnología (CEB).

### Study Site

**Figure 1:**
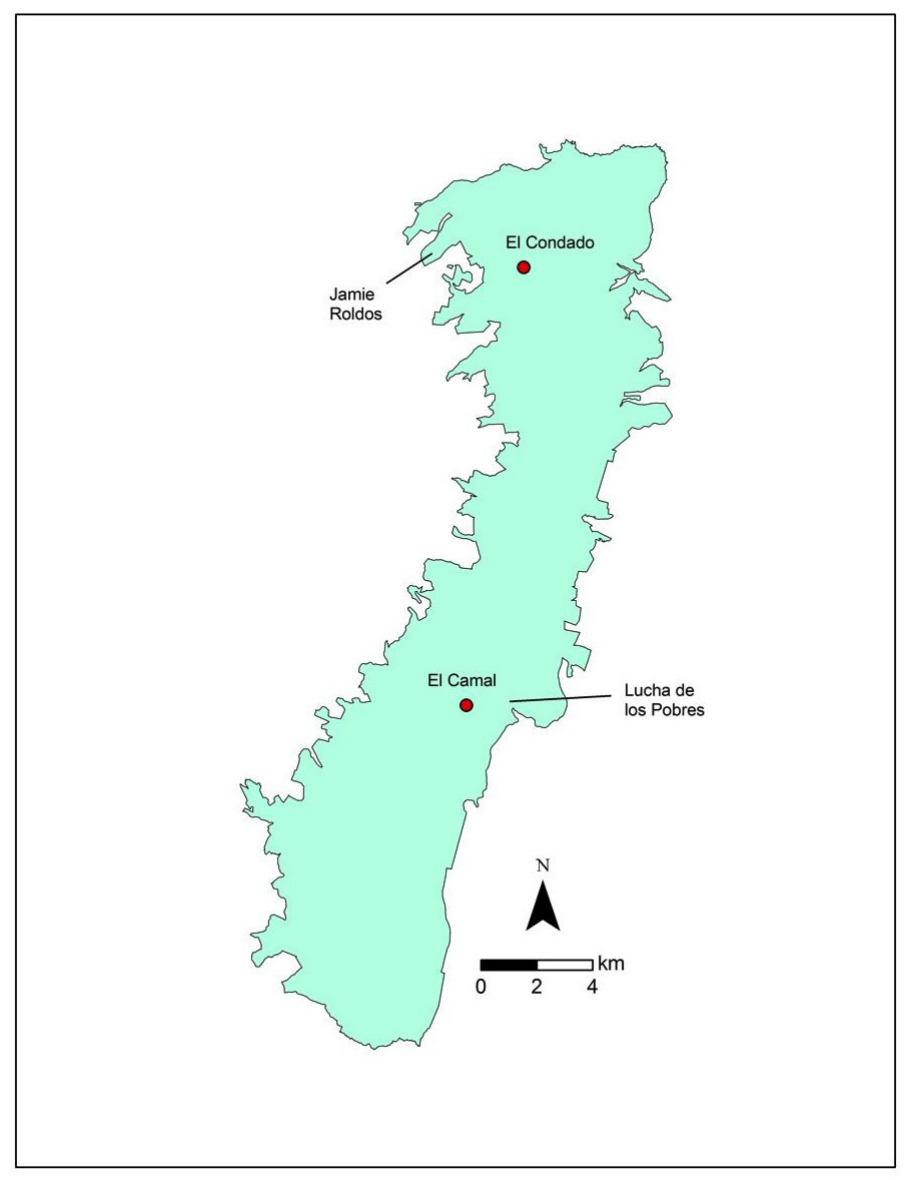
**Map of Quito showing the two neighborhoods (Jaime Roldos and Lucha de los Pobres) where the study was conducted**. El Candado and El Camal are sites adjacent to the two study areas where hourly air monitoring data were collected by CORPAIRE during 2005.

Quito is a high-altitude (2830 m) city with significant ambient air pollution largely due to vehicular traffic and combustion [16]. The city is flanked by volcanic mountains that act to trap air pollutants by reducing atmospheric mixing [17]. Quito's urban slums have high rates of pneumonia and malnutrition [15,18,19]. We identified two neighborhoods, Jaime Roldos (JR) and Lucha de los Pobres (LP), of closely comparable socioeconomic status (SES) (Figure 1, Table 1), but which differ in levels of ambient air pollution. The Instituto Nacional de Estadistica Y Censos (INEC) provided SES and census data for 2001 and the Corporación Para el Mejoramiento del Aire de Quito (CORPAIRE) provided hourly air pollution measurements for 2005 for two monitoring stations located adjacent to the studied neighborhoods.

**Table 1:** Socioeconomic indicators in LP (higher air pollution) and JR (lower air pollution)

Indicator	LP	JR	OR (95%CI)	P-value
Population	11751	9009		

Area (hectares)	95	74		

Male	5888	4451	1.03 (0.97-1.09)	0.32

Female	5863	4558	0.97 (0.92-1.03)	0.32

Illiterate (%)	5.3	5.2	1.02 (0.90-1.16)	0.73

Education^a^				

None (%)	37.7	36.6	0.97 (0.83-1.13)	0.72

Primary (%)	37.7	36.6	1.05 (0.99-1.11)	0.10

Secondary (%)	37.7	37.9	1.05 (0.99-1.11)	0.10

Superior and above (%)	4.3	4.0	1.08 (0.94-1.24)	0.28

Household electricity (%)^b^	98.0	98.0	1.00 (0.82-1.22)	0.99

Absence of poverty (%)	4.7	5.2	0.90 (0.79-1.02)	0.14

Working age population (%)	72.6	72.5	1.01 (0.94-1.07)	0.88

Employment by economic sector (persons)^c^			χ^2 ^= 3.23 (2df)	0.20

Primary	125	87		

Secondary	1437	1226		

Tertiary	3296	2633		

Data provided by INEC. OR, χ^2^, and P-values indicate differences between higher and lower air pollution neighborhoods.

^a^Educational achievement as reported by INEC; unknown or unreported status for 16.9% of residents of LP and 18.0% of JR.

^b^Based on 3231 households in LP and 2379 households in JR.

^c^Primary sector = agriculture, secondary sector = industry, tertiary sector = services.

81 of 8885 individuals were not assigned by INEC to employment sectors.

### Study Design

Between February and May 2006 we enrolled 408 and 413 children aged 18-42 months in LP and JR, respectively, after using a neighborhood census to identify eligible children. For each child we obtained a medical history using a standardized, pre-tested questionnaire for parents asking if the child was hospitalized or received medical attention for a respiratory illness in the prior year, including diagnoses and treatments. Anthropometric measurements included height, weight, and mid-upper arm circumference (MUAC). Length (age < 24 months) or height (age ≥24 months) was measured (nearest millimeter) using a nondistensible plastic tape glued to a rigid board. Weight was measured (closest 0.1 kilogram) using Detecto^® ^Health-o-meters balances and scales (Webb City, IA, USA). MUAC was measured using a nondistensible plastic tape (closest millimeter). Height and weight were converted into weight-for-age (WAZ) and height-for-age (HAZ) Z-scores using Centers for Disease Control (CDC) reference values in the EpiInfo software package [20]. A sterile lancet was used to draw fingerstick blood into a microcuvette analyzer (Hemocue AB, Angelholm, Sweden). Pulse-oximetry blood hemoglobin oxygen saturation (SpO_2_) was assessed via an index finger sensor (opposite hand from fingerstick) after sensor readings stabilized. Anemic children were offered iron supplements. Altitude was measured at each house using a GPS receiver (Garmin, Olathe, KS, USA).

### Analyses

Statistical analysis was performed using EpiInfo (CDC, EpiInfo for Windows, version 3.4.1) and SPSS for Windows (SPSS Incorporated, SPSS Release 14.0). Demographic and illness data were compared using Pearson χ^2 ^analysis and Fisher's exact test. Logistic regression models were used for the multivariate analyses of both outcomes: hospitalization due to pneumonia and outpatient consultations due to acute respiratory illness (ARI). Associations were assessed by calculation of the odds ratio (OR) with 95% confidence intervals (CI). Following international conventions, children with height for age (HAZ) z-scores ≤ 2 standard deviations below the mean age and gender adjusted means were termed stunted. Anemia was defined as an adjusted blood hemoglobin < 11.0 g/dL after subtracting 1.9 g/dL to adjust for altitude [21]. In order to quantitatively check our assumption of significant air pollution differences by neighborhood, median air pollution levels were tested using the Mann-Whitney test.

## Results

**Figure 2:**
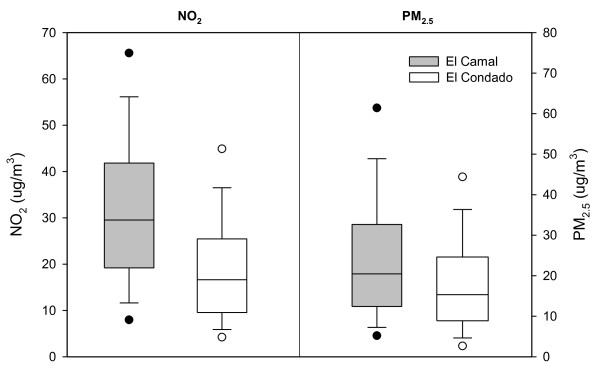
**Median PM_2.5 _in μg/m^3 ^and median NO_2 _in μg/m^3 ^during 2005 at the air pollution monitoring sites adjacent to the study neighborhoods**. Median PM_2.5 _and NO_2 _levels were each significantly lower (P < 0.001) at El Condado than at El Camal. El Condado is adjacent to JR, the lower air pollution site, and El Camal is adjacent to LP, the higher air pollution site.

There were no significant differences in population density, educational achievement, illiteracy, overall poverty, and other SES indicators between Lucha de los Pobres and Jamie Roldos (Table 1). There was a total of 7808 hourly NO_2 _measurements from LP and 8065 from JR, while for PM_2.5 _there were 8449 measurements from LP and 6157 from JR. The annual median NO_2 _level for LP was 29.5 μg/m^3 ^and for JR it was 16.6 μg/m^3^. The annual median concentration of PM_2.5 _for LP was 20.4 μg/m^3 ^and for JR it was 15.3 μg/m^3^. Box plots of the data are shown in Figure 2. The nonparametric Mann-Whitney test for equal median levels demonstrated highly significant (P < 0.001) differences for both pollutants. We considered Lucha de los Pobres to be a community with relatively higher air pollution levels compared to Jamie Roldos, a community with relatively lower air pollution levels.

Complete demographic and anthropometric data were available for 798 (97%) children (Table 2). Neither enrollee age nor gender proportions differed by neighborhood. Children in LP were significantly more malnourished (lower HAZ, WAZ, and hemoglobin) than children in JR (Table 2). Likewise, stunting and anemia were significantly more prevalent in LP children than in JR children (Table 2), with odds ratios (ORs) of 1.88 and 1.45, respectively.

**Table 2:** Demographic, nutritional, and environmental information for children exposed to higher (LP), and lower (JR), air pollution.

	LP	JR	
	**N = 401**	**N = 397**	

	**mean (sd)**	**mean (sd)**	**P**

**Demographic characteristics**			

Age (months)	31.25 (7.51)	31.25 (7.51)	0.94

Males (number, %)	209 (50.6)	224 (54.5)	0.26

**Nutritional characteristics**			

HAZ (height-for-age)	-1.46 (1.00)	-1.28 (0.87)	0.006

Presence of stunting^a^	125/397	78/401	< 0.001

WAZ (weight-for-age)	-1.31 (1.09)	-1.12 (0.97)	0.010

MUAC (cm)	15.44 (1.09)	15.43 (1.07)	0.900

Adjusted hemoglobin (g/dL)^b^	10.45 (1.39)	10.74 (1.14)	0.002

Presence of anemia^c^	253/401	214/397	0.013

**Environmental measures**			

SpO_2 _(%)	92.2 (2.60)^d^	95.8 (2.20)	< 0.001

Mean household altitude (m)	2979 (72)^e^	2852 (47)	< 0.001

P-values represent the significance level of t-test comparisons unless otherwise noted.

^a^Stunting was defined as a HAZ *z-*score ≤ -2 (i.e., 2 standard deviations below the mean age- and sex-adjusted height).

^b^Hemoglobin was adjusted for altitude by subtracting 1.9 g/dL from the measured value [21].

^c^Anemia was defined as an adjusted blood hemoglobin < 11.0 g/dL.

^d^n = 400 for the high exposure children (LP), and 397 for lower exposure children (JR).

^e^n = 398 for the high-exposure children (LP), 397 for lower exposure children (JR)

**Figure 3:**
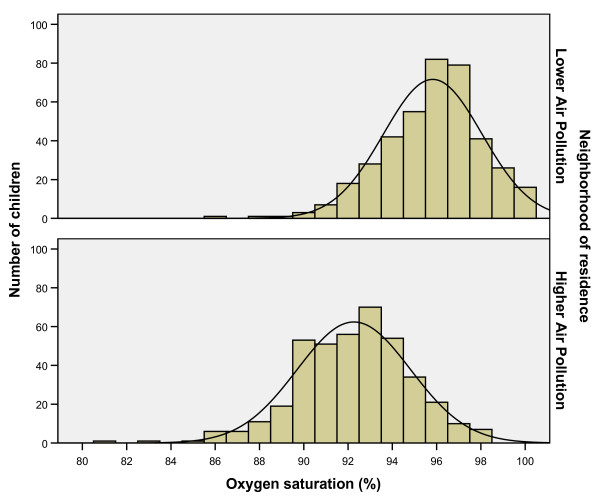
**Pulse-oximetry oxygen saturation (SpO_2_) values by residential ambient air pollution exposure**. SpO_2 _are significantly lower in the high air pollution neighborhood, compared to the lower air pollution neighborhood, P < 0.001.

High exposure children had significantly lower SpO_2 _than low exposure children (92.2% ± 2.6% vs. 95.8% ± 2.2%; P < 0.001; Figure 3). The high exposure households were at a greater mean altitude than the low exposure households (2979 ± 79 versus 2852 ± 47 m, P < 0.001), corresponding to 527.2 and 535.7 Torr of atmospheric pressure, respectively. However, this minor pressure difference is not enough to account for the SpO_2 _difference [22].

### Hospitalizations due to pneumonia, physician consultations for ARI, and air pollution exposure

All 22 hospitalizations were for pneumonia. Of the hospitalized children, 7 resided in the lower air pollution exposure neighborhood while 15 resided in the higher air pollution exposure neighborhood. Parents were able to recall a specific diagnosis for 97% of physician consultations for all ailments in 2005 and this percentage did not vary between neighborhoods. The most common outpatient diagnoses were asthma, bronchitis, cough, influenza, pharyngitis, pneumonia, sinusitis, and tonsillitis. A total of 172 of 397 children (43%) with lower air pollution exposure required one or more consultations versus 224 of 401children (56%) with higher air pollution exposure.

Children with higher air pollution exposure were significantly more likely to have had a pneumonia hospitalization (Table 3) or ARI requiring physician consultation (Table 4) than children exposed to lower air pollution exposure. In logistic regression analysis controlling for age, gender, HAZ, WAZ, hemoglobin, and residential altitude, higher air pollution exposure was significantly associated with pneumonia hospitalization (OR = 3.68, 95% CI 1.09-12.44; P = 0.036; Table 3).

**Table 3:** Hospitalization for pneumonia: Logistic regression analysis controlling for cofactors.

	All children (N = 792)			Not anemic (N = 331)			Anemic (N = 461)		
**Cofactors**	**OR**	**95%CI**	**P**	**OR**	**95%CI**	**P**	**OR**	**95%CI**	**P**

Age	1.11	1.03-1.19	0.004	1.06	0.94-1.18	0.36	1.14	1.04-1.26	0.007

Gender	1.02	0.43-2.44	0.96	0.60	0.13-2.90	0.52	1.35	0.46-4.02	0.59

HAZ	1.48	0.76-2.89	0.25	1.50	0.46-4.85	0.45	1.54	0.66-3.57	0.32

WAZ	0.86	0.48-1.54	0.60	0.81	0.30-2.22	0.69	0.83	0.40-1.73	0.63

Hemoglobin	0.92	0.65-1.31	0.65	0.63	0.20-1.97	0.43	0.76	0.38-1.54	0.45

Air pollution exposure*	3.68	1.09-12.4	0.036	1.04	0.08-13.9	0.98	6.82	1.45-32.0	0.015

Altitude	1.00	0.99-1.01	0.21	0.99	0.98-1.01	0.25	1.01	1.002-1.02	0.017

**Table 4:** Medical consultations for respiratory illness: Logistic regression analysis controlling for cofactors.

	All children (N = 792)			Not anemic (N = 331)			Anemic (N = 461)		
**Cofactors**	**OR**	**95%CI**	**P**	**OR**	**95%CI**	**P**	**OR**	**95%CI**	**P**

Age	1.01	0.99-1.03	0.23	1.02	0.99-1.05	0.26	1.01	0.98-1.04	0.49

Gender	1.04	0.78-1.38	0.81	0.86	0.52-1.28	0.38	1.24	0.85-1.80	0.26

HAZ	0.97	0.79-1.20	0.78	0.86	0.61-1.20	0.60	1.08	0.82-1.42	0.58

WAZ	0.94	0.78-1.14	0.53	1.09	0.80-1.48	0.60	0.84	0.65-1.08	0.63

Hemoglobin	1.08	0.96-1.20	0.22	1.01	0.71-1.42	0.97	1.11	0.88-1.39	0.38

Air pollution exposure*	2.93	1.92-4.47	< 0.001	3.28	1.72-6.24	< 0.001	2.66	1.50-4.70	0.001

Altitude	1.004	1.002-1.007	0.001	1.004	1.001-1.008	0.022	1.004	1.001-1.007	0.017

There were no significant differences in age, gender, or nutritional parameters (HAZ, WAZ, MUAC, hemoglobin) between previously ill and healthy children. However, SpO_2 _was significantly lower in previously ill children compared to healthy children (93.6 ± 3.0% vs. 94.5 ± 3.0%; P < 0.001). In chi-square and regression analyses, neighborhood SES factors were not associated with any nutritional, environmental, or illness characteristics or outcomes.

### Modification of the effect of air pollution exposure by anemia

Fifteen of the 22 hospitalized children were anemic. The presence of anemia increased the OR for pneumonia hospitalization in children with high air pollution exposure from 3.68 to 6.82 (Table 3). In the absence of anemia, higher air pollution exposure was not predictive of pneumonia hospitalization (OR 1.04; 95% CI 0.08-13.93). In contrast, anemia status did not change the influence of air pollution exposure on ARI events that required consultation with a physician (Table 4).

## Discussion

Our most important result is that anemic children exposed to relatively high levels of ambient air pollution are at increased risk of pneumonia hospitalization compared to children exposed to lower levels of air pollution. In addition, we found that children (both anemic and non-anemic) who were exposed to higher levels of air pollution were significantly more likely to have received a physician consultation for acute respiratory illness compared to children exposed to lower levels of air pollution. We also found evidence that anemia and higher air pollution exposure may interact to increase the odds of hospitalization in children with pneumonia. The strongest evidence of the potential interaction effect is that anemia increased the OR for the association between higher air pollution exposure and pneumonia hospitalization from OR (3.68, 95% CI 1.09-12.44; P = 0.036) for all children to OR 6.82 (95% CI 1.45-32.00; P *= *0.015) for anemic children. In non-anemic children, the OR for hospitalization with pneumonia in children exposed to higher levels of air pollution dropped from 3.68 (noted above) to 1.04 (95% CI 0.08-13.9; P = 0.98). These results suggest a possible interaction between anemia and ambient air pollution exposure as risk factors. This result is important as these risk factors have not been examined concurrently before, however, our study did not have the statistical power to formally assess the potential interaction between pneumonia hospitalization and anemia status. Larger studies specifically designed to examine this finding are required.

The central pathophysiological deficit in pneumonia is poor tissue oxygenation [23]. Anemia and low hemoglobin oxygen saturation independently decrease oxygen delivery. Hypoxia increases mortality during pneumonia two to five-fold [23,24]. The association between anemia and pneumonia has been recognized since at least 1925 [25]. Anemic children have less oxygen carrying capacity-and suffer greater hypoxia during pneumonia-than normal children [26]. Oxygen saturation was significantly lower (P < 0.001) in children with high air pollution exposure. It is biologically plausible that anemia, and the lower hemoglobin oxygen saturation of higher air pollution exposure children, could additively or multiplicatively decrease tissue oxygenation during pneumonia, especially at high altitude, resulting in more severe disease and more hospitalizations.

Reasons for differential oxygen saturations in normal populations are limited to only two possibilities: either marked differences in altitude, which was not the case in our study (127 m difference), or dyshemoglobinemia [26]. Carbon monoxide (CO) and NO_x _(particularly NO), produced by vehicular engine combustion, reduce oxygen saturation through formation of the dyshemoglobins carboxyhemoglobin and methemoglobin [27]. Vehicular engine combustion is the major source of air pollution in Quito [12,16,17].

Previously, Estrella et al. [12] documented elevated carboxyhemoglobin levels in primary school children in Quito. Carboxyhemoglobin levels were 2.52% ± 1.12 in a northern Quito school adjacent to our lower pollution neighborhood, and 5.09% ± 1.7 (P < 0.05) in a central city school, adjacent to our higher air pollution neighborhood. Carboxyhemoglobin levels above 2.5% are abnormal; 43% of children living in northern Quito and 92% of the children living in central Quito exceeded this threshold [12]. Rare causes of dyshemoglobinemia (anesthetic or dapsone use, high-nitrate water ingestion), are unlikely alternative explanations since few children in this study would have undergone anesthesia or dapsone treatment, and all children shared a low-nitrate municipal water supply. Therefore, the most likely explanation for the marked SpO_2 _difference between the higher and lower air pollution exposure children is their differential exposure to common, combustion-related, air pollutants that bind to hemoglobin.

One potential limitation of our O_2 _saturation data was that we used non-invasive pulse oximetry rather than directly measuring carboxyhemoglobin and methemoglobin levels, as the latter would have required venous blood sampling, potentially decreasing study participation. However, oximetry underestimates carboxyhemoglobin-related oxygen saturation deficits [26,27], suggesting the deficits we found are conservatively estimated.

This study supports interaction between ambient air pollution levels, anemia, and hospitalization for pneumonia because (1) the OR increased in the setting of both risk factors, and (2) a biologically plausible pathophysiological mechanism for synergy can be identified. However, our study was designed for a straightforward comparison and sample size was too small to conclusively test for significant interaction in either tabular analysis using the Breslow-Day approach or by using an interaction term in the logistic regression model. Mauderly and Samet (2009) noted the difficulty of establishing synergism in air pollution studies because sample sizes of people with impaired health are generally too low for rigorous statistical testing [28]. This study, however, is informative for the design of larger studies looking specifically for evidence of this interaction.

The study children resided in impoverished neighborhoods (Table 1) which were carefully chosen to minimize differential nutritional status secondary to family socioeconomic status. We hypothesize that the incrementally worse nutritional status (HAZ, WAZ, and anemia) of the higher exposure children (Table 2) was due to their incrementally higher incidence of respiratory infections related to higher air pollution (Tables 3 and 4). Higher rates of infection are known to worsen nutritional status, including anemia, in children [1,2,10,11,29].

It could be argued that the increased pneumonia hospitalization of the higher air pollution exposure children was due to their greater malnutrition, and not their air pollution exposure. However, in the entire study population, children with and without a history of respiratory illness did not differ by age, gender, or any nutritional parameters (HAZ, WAZ, MUAC, hemoglobin). The only significant difference between ill and healthy children was the lower oxygen saturation level seen in children with a pneumonia hospitalization or physician consultation (93.6 ± 3.0% vs. 94.5 ± 3.0%; P < 0.001). We believe this difference in SpO_2 _represents air pollution-related dyshemoglobinemia.

Data from a retrospective study must be interpreted cautiously. First, although the site populations did not differ by SES metrics, we did not collect household SES data that potentially could have provided additional insight into the role(s) of various socioeconomic factors. Second, families had to recall health information regarding their children over the previous 12 months. Although we inquired about events that were likely to be remembered (indeed, specific diagnoses were recalled for all (100%) hospitalizations and 495 of 512 (97%) physician consultations), we could not independently verify that all recalled diagnoses were accurate. Third, we did not correct for some potential confounding variables such as biofuel smoke in indoor air or cigarette smoking, however, ~ 98% of households in both sites used gas and not biofuels (Table 1), and therefore it is unlikely that biofuel smoke introduced a major source of bias. Also, in a tobacco-use survey (Harris et al, unpublished data), 30% of adults in LP and JR stated that they smoked cigarettes but 96% of users smoked ≤5 cigarettes per day because of cost. Furthermore, Estrella et al [12] found no difference in carboxyhemoglobinemia in children from households with and without tobacco use. These factors suggest that smoking was not a major source of bias in our study. We used data for 2005 from CORPAIRE, which monitors ambient air pollution using accepted methods [17,30] and we assumed that living in a more polluted neighborhood was linked to higher personal exposure to ambient air pollution, a widely accepted assumption [6]. Lastly, it is possible that unknown confounding factors we did not measure accounted for the higher rates of pneumonia, rather than the combination of air pollution and anemia.

In summary, we found evidence of possible synergy between a common manifestation of malnutrition-anemia-and ambient air pollution as risk factors for pneumonia hospitalization in a high-altitude setting. Their effects on oxygen delivery could plausibly mediate this association. We suspect air pollution-related dyshemoglobinemia reduces oxygen saturation, while anemia decreases oxygen carrying capacity. Our study suggests that both improving nutrition, and controlling air pollution, could most rapidly decrease hospitalizations for pneumonia, the leading cause of death in children. Further study is needed to confirm our finding of synergy between air pollution and anemia.

## Competing interests

The authors declare that they have no competing interests.

## Authors' contributions

AMH, FS, and BE contributed to study design, data collection, data analysis, and manuscript preparation. XN, and JE contributed to data collection and manuscript preparation. MW, JLD, and ENN contributed to data analysis and manuscript preparation. JKG contributed to study design, data analysis, and manuscript preparation. All authors read and approved the final manuscript.
